# *Plasmodium falciparum* Transcriptome Analysis Reveals Pregnancy Malaria Associated Gene Expression

**DOI:** 10.1371/journal.pone.0001855

**Published:** 2008-03-26

**Authors:** Nicaise Tuikue Ndam, Emmanuel Bischoff, Caroline Proux, Thomas Lavstsen, Ali Salanti, Juliette Guitard, Morten A. Nielsen, Jean-Yves Coppée, Alioune Gaye, Thor Theander, Peter H. David, Philippe Deloron

**Affiliations:** 1 Institut de Recherche pour le Développement (IRD), UR010 at Université Paris Descartes, Mother and Child Health in the Tropics, Faculté de Pharmacie, Paris, France; 2 Institut Pasteur, Plate-Forme 2 - Puces à ADN, Paris, France; 3 Centre for Medical Parasitology at University of Copenhagen, Copenhagen, Denmark; 4 Centre de Santé Roi Baudoin de Guediawaye, Dakar, Senegal; 5 Institut Pasteur, URA CNRS 2581 Unité d'Immunologie Moléculaire des Parasites, Paris, France; Walter and Eliza Hall Institute of Medical Research, Australia

## Abstract

**Background:**

Pregnancy-associated malaria (PAM) causing maternal anemia and low birth weight is among the multiple manifestations of *Plasmodium falciparum* malaria. Infected erythrocytes (iEs) can acquire various adhesive properties that mediate the clinical severity of malaria. Recent advances on the molecular basis of virulence and immune evasion have helped identify *var2csa* as a PAM-specific *var* gene.

**Methodology/Principal Findings:**

The present study presents a genome-wide microarray transcript analysis of 18 *P. falciparum* parasite isolates freshly collected from the placenta. The proportion of PAM over-expressed genes located in subtelomeric regions as well as that of PAM over-expressed genes predicted to be exported were higher than expected compared to the whole genome. The identification of novel parasite molecules with specificity to PAM and which are likely involved in host-pathogen interactions and placental tropism is described. One of these proteins, PFI1785w, was further characterized as the product of a two-exon PHIST gene, and was more often recognized by serum samples from *P. falciparum*-exposed women than from men.

**Conclusions/Significance:**

These findings suggest that other parasite proteins, such as PFI1785w, may contribute beside VAR2CSA to the pathogenesis of PAM. These data may be very valuable for future vaccine development.

## Introduction

Clinical severity of *Plasmodium falciparum* malaria is linked in part to alterations of the infected erythrocyte (iE) induced by parasite proteins exported to the iE membrane during blood stage development. Some of these proteins confer cytoadherence properties to the iE, leading to parasite sequestration from the blood stream. The best characterised parasite adhesin is *P. falciparum* erythrocyte membrane protein 1 (PfEMP1), encoded by the large polymorphic *var* gene family. This adhesin undergoes antigenic variation, a phenomenon that is thought to contribute to parasite evasion from the host immune response [1]. Certain relatively conserved sub-families of PfEMP1 molecules have been associated to differences in disease outcome [2], [3], [4].

Malaria during pregnancy and particularly first pregnancies is associated with dramatic adverse effects on fetal growth (reviewed in [5]). Primigravidae lack protective antibodies, which suggests that pregnancy-associated malaria (PAM) parasites express novel surface molecules to which women have not been exposed previously [6], [7], [8], [9]. PAM parasites show placental tropism and specifically adhere to chondroitin sulphate A (CSA) [6], [10]. Plasma inhibitory activity to iE binding CSA is not dependent on the geographical origin of parasites or plasma [7], indicating that proteins mediating placental tropism are conserved between parasite genotypes or contain shared epitopes and that a vaccine against this form of malaria can be developed.

PfEMP1 proteins encoded by parasite *var* genes and expressed on the surface of iEs were extensively shown to mediate various binding activities hence representing an important target for antibodies associated with protective immunity against malaria. Several studies have shown that a single *var* gene, *var2csa*, is highly transcribed and expressed in both *in vitro* CSA panned *P. falciparum* iEs and parasites from infected placentas [4], [11], [12], [13], [14]. The finding that antibody levels to VAR2CSA are dependent on sex and gravidity and are associated with reduced consequences of PAM [15], [16] has further strengthened the candidacy of VAR2CSA as the CSA adhesin in PAM. However, other genes may be involved either in facilitating the VAR2CSA expression and binding or in other unknown PAM associated mechanisms. Identification of these putative genes would be crucial for our understanding of PAM and the development of a vaccine against this particular form of malaria.

In the present study, DNA microarray analysis of genome-wide expression profiles from 18 fresh *P. falciparum* isolates directly collected from human placentas at delivery allowed the identification of novel genes associated with placental tropism.

## Results

### Identification of differentially transcribed genes in placental parasites compared to 3D7 reference

To assess the steady-state of mRNA levels of *P. falciparum* infecting pregnant women, parasites were collected from the placenta of delivering women in Senegal. Microscopic examination demonstrated a domination of late stage iEs (trophozoites and shizonts) in all isolates. Due to the variable distribution of late stages, samples were divided into 3 groups: (pool 1 included 3 isolates: ≤5% rings, 70% trophozoites, ≥25% schizonts; pool 2 with 7 isolates: ≤5% rings, ≥25% trophozoites, 70% schizonts; pool 3 with 8 isolates: ≤5% rings, 0% trophozoites, ≥95% schizonts). Total RNA was isolated from each individual sample and RNA from individuals corresponding to each defined group was pooled by mixing equal quantities of RNA. We chose to explore placental parasite gene expression versus a common 3D7 reference and preferentially select the genes differentially expressed in all 3 groups of placental parasites. Reference parasite population was made-up of 10% rings, 45% trophozoites and 45% schizonts. RNA pooled samples were fluorescently-labeled prior to hybridization to an array of high-density oligonucleotides.

**Figure 1:**
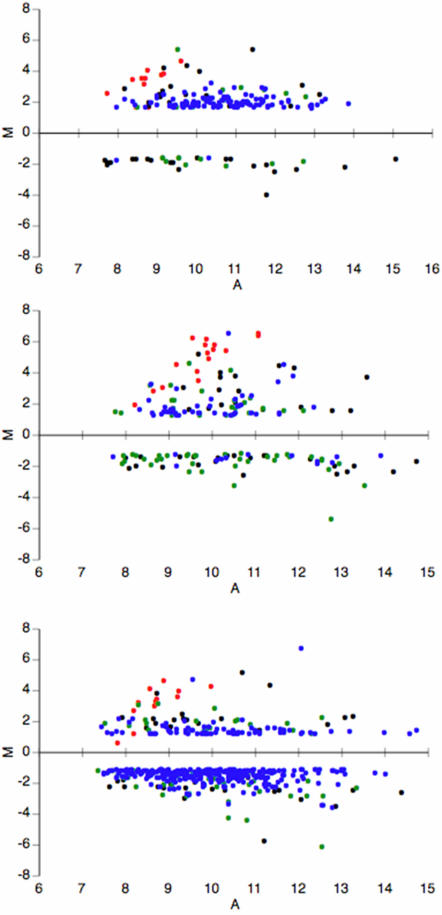
Transcriptional characteristics of placental isolates. Plots of log2 ratio of expression (M) against average log intensity (A) for three different pools of placental isolates (pool 1 included 3 isolates: ≤5% rings, 70% trophozoites, ≥25% schizonts; pool 2 with 7 isolates: ≤5% rings, ≥25% trophozoites, 70% schizonts; pool 3 with 8 isolates: ≤5% rings, 0% trophozoites, ≥95% schizonts) versus common 3D7 reference (10% rings, 45% trophozoites, 45% schizonts). Only statistically significant data according to Bonferroni p value correction are shown. A single dominant *var* gene (*var2csa*) is detected in placental isolates of all groups examined (red dots; each dot represents one oligonucleotide). Non *var* genes over-expressed or under-expressed are shown as black (when the difference was present in all 3 pools), green (in 2 of the 3 pools). Blue dots correspond to oligonucleotides corresponding to genes only differentially expressed in 1 out of 3 experiments (these were not taken into account in our analysis).

Using the Bonferroni adjustment method, 84 genes consistently displayed similar differential expression profiles in 2 out of 3 groups (over-expressed N = 38; under-expressed N = 46) (Figure 1, Table 1). These genes generally had highly abundant transcripts either in the test samples or in the 3D7 reference pool as judged by spot fluorescence values. *Var2csa* was the only *var* gene unambiguously over-expressed. Over-expression in placental parasites was observed in 14 out of 18 different *var2csa* oligonucleotides (data not shown). Other *var* hybridising probes showing transcript abundance differentials represented either truncated or pseudo-genes or *var* exon2, [17].

**Table 1:** Total number of genes that were differentially expressed in in vivo parasite isolates compared with expression in in vitro 3D7 reference.

	2 of 3		3 of 3		total	
	up	down	Up	down	up	down
**Oligos**	28	30	27	17	55	47
**Genes**	19	30	19	16	38	46
**Hypothetical protein**	6	19	7	8	13	27

The number of oligos used and the number of those genes encoding hypothetical or predicted exported proteins are also shown.

**Figure 2:**
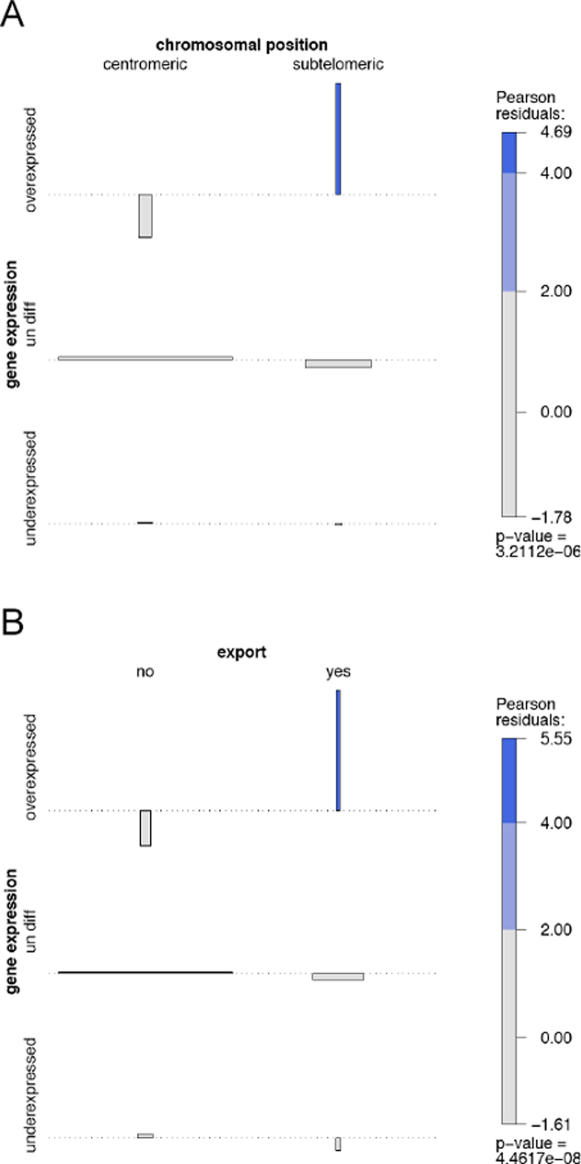
Association plot between gene expression and, (A) chromosome location, (B) predicted host cell export. Bar width indicates the number of genes within each category while bar high indicates deviations from an independent model showing categories contributing to the disequilibrium. Result of chi2 test is indicated above the legend. *Multi-copy gene families such as *var, rif, stevor* and *Pfmc-2TM* were excluded from the data used to calculate the bias.

The proportion of genes in subtelomeric regions over-expressed by placental parasites was higher than expected compared to the whole genome (p = 3.2e-6) (figure 2, panel A); no such bias was observed with placental parasites under-expressed genes. Similarly, the proportion of over-expressed genes predicted to be exported [18]was higher than expected compared to the whole genome (p = 4.5e-8) (figure 2, panel B), whereas no bias was observed with placental parasites under-expressed genes.

### Validation of microarray data by real-time PCR

To confirm the accuracy of the microarray results, the transcript abundance of 13 selected genes was carried out. The genes were selected based on over or under-expression, abundance of transcripts and the presence of at least one TM, export prediction, and/or signal sequence motifs (see supplement table S1). Real-time PCR was performed first with the three pooled RNA/cDNA samples used in the microarray analysis, then on RNA/cDNA from each individual parasite sample included in the pool. Overall, real-time PCR confirmed the arrays (Spearman correlation test after log-transformation of the data, r = 0.79, p<.0001) (**Table 2**).

**Table 2:** Confirmation by real-time quantitative rt-PCR of in vivo expression of mRNA for 13 different genes, compared with that in 3D7 in vitro reference.

Microarray fold change			Gene ID	rt-qPCR fold change		
Group 1	Group 2	Group 3		Group 1	Group 2	Group 3
25,03	90,27	18,48	PFL0030c (+)	40,78	99,76	109,67
40,97	21,55	19,88	PFC0110w (+)	10,66	18,4	23,15
7,08	35,45	4,63	PFI1785w (+)	149,15	319,69	206,25
5,5	12,89	3,43	PF10_0351 (+)	5,56	6,63	5,03
5,61	12,74	3,1	PFA0700c (+)	103,7	171,37	112,99
5,61	3,74	3,54	PF14_0010 (+)	10,2	26,48	5,75
8,36	3,39	2,69	PF10_0344 (+)	2,76	3,81	1,91
3,31	2,93	4,59	PF14_0757 (+)	2,84	1,63	0,81
6,72	2,55		PFB0105c (+)	3,95	4,4	4
			PF13_0304 ( = )	0,94	1,2	0,72
	0,34	0,23	PFL0260c (−)	0,16	0,22	0,28
	0,32	0,17	PF10_0350 (−)	0,56	0,61	0,26
0,3	0,3	0,16	P FD1120c (−)	0,26	0,22	0,19

Gene's selection criteria were based on similar expression observed in at least 2 out 3 tests sample pools. Values are fold changes calculated as compared to the 3D7 level. Gene expression levels ranged from overexpressed in *in vivo* parasites to fairly expressed according to arrays measurements. (+) For over-expressed in placental parasites according to array data and (−) For under-expressed in placental parasites according to array data. One gene with no difference in expression ( = ) according to microarray was also included. Empty cells correspond to no difference observed.

### Biological validation of array data

**Figure 3:**
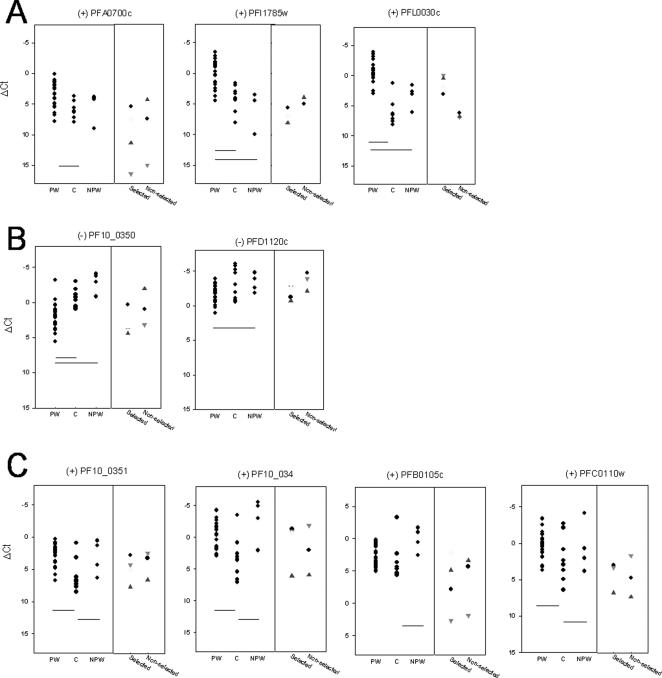
Transcription levels measured by real-time qPCR in parasites from different clinical malaria status. Blocks A to C, left panels correspond to higher transcription levels in: (A) placental parasites from pregnant women at delivery (PW); (B) non placental parasites from children (C) and non placental parasites from non pregnant symptomatic women (NPW); (C) parasites from women (PW or NPW). Shown are transcription levels for 22 parasites isolated from the placenta of women (PW), 9 parasites from asymptomatic children (C) (mean age 10 years) and 4 parasites from symptomatic non-pregnant women (NPW) living in the same area, as well as by (in the right side of each panel) the *in vitro* cultured *P. falciparum* strains 3D7 (black dots), FCR3 (red dots), and NF54 (blue dots). Each of these 3 strains were used either non-selected or after selection for CSA binding using panning on soluble CSA (3D7), Bewo cells (FCR3), or VAR2CSA IgGs (NF54). Hb3 (yellow dots) was also studied after selection on Bewo cells. These parasite lines were highly synchronized and aged 24 hours post invasion Missing points correspond to lack of detection of the corresponding gene (in particular for PFI1785w in FCR3). Horizontal bars indicate statistically significant differences between groups. On top, (+) indicates over-expressed in PAM parasites according to array data; (−) indicates under-expressed in PAM parasites according to array data.

To further assess biological relevance of the microarray findings, the transcription abundance of 13 genes selected for real-time PCR was determined in parasites associated with different malaria clinical backgrounds. Transcription abundance of the 13 genes was also measured in laboratory-adapted parasite lines selected or not for PAM phenotypes. By examining the transcription profiles of parasites from different clinical groups, four categories of transcription profiles were defined: (A): highest expression in placental parasites (B): highest expression in parasites from symptomatic women and asymptomatic children (C): highest expression in parasites from symptomatic women and pregnant women but not from asymptomatic children (**Figure 3**) and no expression difference among parasites from all 3 clinical groups (**not shown**). Overall 9 of the 13 tested genes behaved as predicted regarding differential expression between parasites from PAM and non-PAM donors. In group A, in addition to *var2csa*, one gene, PFI785w, encoding a hypothetical protein displayed a placental parasite-specific profile similar to that of *var2csa.* PFI1785w encodes a 39.4 kD protein that is predicted to be exported to the red blood cell (**table S2**). In group B (**Figure 3**), the two RT-PCR tested genes found to be PAM under-expressed by microarray analysis, PFD1120c and PF10_0350 were also found under-expressed in placental parasites when compared to non pregnant women and children parasites. In group C, the four genes explored were over-expressed in symptomatic adults compared to asymptomatic children.. Microarray detected PAM over-expression of PF14_0757 was not confirmed by real-time qPCR. However, although microarray data were confirmed by RT-PCR for PF14_001, PFL0260c, no differential expression was observed among the different groups of clinical isolates analyzed.

When transcript abundance of the 13 genes was measured in laboratory-adapted parasite lines selected or not for CSA binding phenotype (**Figure 3, left panel**), only *var2csa*, displayed a marked specificity for CSA–binding [13], [19].

### Characterization of PFI1785w

**Figure 4:**
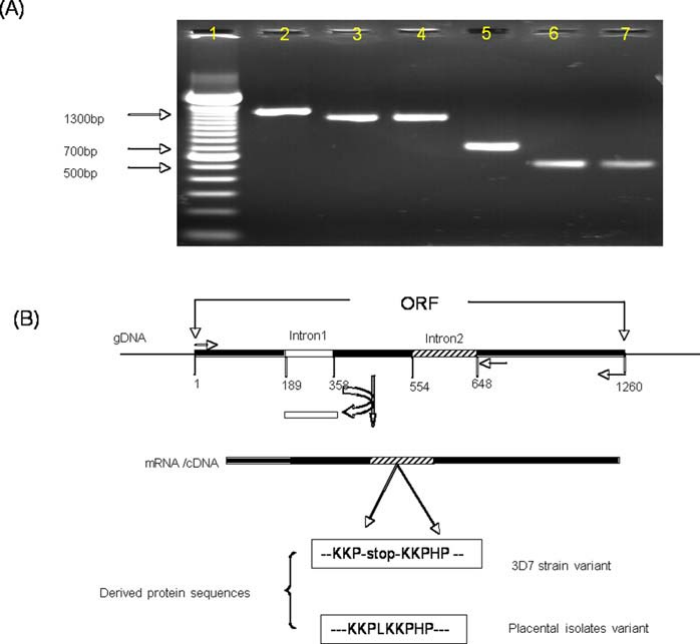
PFI1785w spliced structure. (A) PCR products confirming splicing of intron 1 but not intron 2 on 3D7 and a placental isolate cDNA. Primers indicated on (A) by arrows were used to amplify full size and a short fragment of the gene on 3D7 genomic DNA (lane 2 and 5), and on cDNA of 3D7 (lane 3 and 6) and a placental isolate (lane 4 and 7). Lane 1 indicates a 100 bp DNA lader. (B) Diagram of exon/intron structures from predicted gene and mRNA/cDNA. The heavy bars are predicted exons. Empty and stripped bars are predicted intron 1 and intron 2 respectively according to 3D7 gDNA sequence. Figures below the gDNA structure indicate nucleotide positions. mRNA/cDNA structure is the experimentally observed structure.

The specific over-expression of PFI1785w by fresh placental isolates, and not by non-PAM parasites or by *in vitro* CSA-selected laboratory lines, raised a particular interest for further characterization. An interesting aspect of PFI1785w is the presence of a PHIST (*Plasmodium* helical interspersed subtelomeric family) domain, and thereby a predicted role in erythrocytic trafficking or remodeling [18]. PFI1785w conforms to the canonical gene structure of Pexel-containing proteins in which the signal peptide is encoded by the first exon, and the Pexel motif and the remainder of the protein are encoded on the second exon. However, PFI1785w differs from almost all Pexel-encoding genes in that it possesses a third exon according to the 3D7 annotation in the current parasite database. The PFI1785w genomic sequences amplified in this study confirmed the presence of the in-frame stop codon at aminoacid position 146 in the 3D7 gDNA, which is not present in the alleles from the Ghanaian (Sanger Institute Browser) or HB3 (Broad Institute Browser) strains. However the spliced structure as determined by PCR and sequencing of the cloned 3D7 PFI1785w gDNA and corresponding full size cDNA from 3D7 and from 2 placental isolates revealed existence of only one intron on this gene and the splicing site was defined between nucleotide position 189 and 358 (figure 4). All cDNA sequences data have been submitted to the GenBank with the following accession numbers: EU181220 (for 3D7), EU181221(for placental isolate NTN42), EU181222 (for placental isolate NTN14). The nucleotide sequence was highly conserved between 3D7 and the two placental isolates cDNA. Only two point mutations were observed. Our data indicate that the PFI1785w mRNA was predicted incorrectly. This raises the necessity to confirm the current annotation of the parasite genome with experimental data.

**Figure 5:**
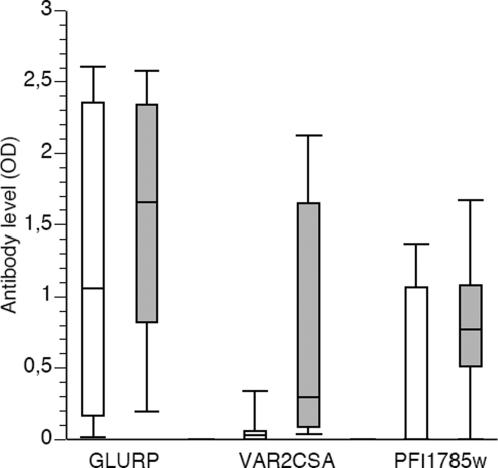
Recognition of PFI1785w recombinant protein (NP561) by sera from malaria exposed individuals assessed by ELISA. ELISA was carried out on NP561, DBL5-ε of VAR2CSA and GLURP coated plates. The levels of IgG expressed as OD values are shown for exposed Ghanaian men (n = 30; white bars) and women (n = 30; grey bars). The top, bottom, and line through the middle of the box correspond to the 75^th^ percentile, 25^th^ percentile, and 50^th^ percentile (median), respectively. The whiskers on the bottom extend from the 10^th^ percentile and top 90^th^ percentile. Plasma concentrations of anti-VAR2CSA and anti-NP561 IgG were significantly higher in the women group.

Part of the corresponding protein encoded by PFI1785w was expressed in *baculovirus* transfected insect cells and purified as a secreted HIS protein (that we called NP561). NP561 was recognized in ELISA by serum samples from individuals living in endemic areas (Ghana). Sera from women had significantly higher levels of antibodies against NP561 compared to sera from men of the same area in Ghana (p = 0.04). Recognition of another parasite recombinant protein (GLURP) was also measured and no significant difference was observed between sera from both genders (**Figure 5**).

## Discussion

Many studies have generated convincing evidence of the unique antigenic and binding properties of *P. falciparum* iEs collected from the placenta. Previous analysis of the parasite variant surface antigens has pointed to PfEMP1 variants as the major target of antigenic variation and mediator of iE adhesion properties. Our prior studies have shown that VAR2CSA PfEMP1 accounts for CSA binding by iEs originating from the placenta [4]. The present study has identified differences in gene expression compared to *in vitro* 3D7 parasite reference, using the whole-genome microarray analysis of *P. falciparum* parasites directly collected from the placenta. First, this analysis confirmed *var2csa* (represented by 16 oligonucleotides) as the only *var* gene clearly over-expressed in all placental parasite isolates. This however does not exclude possible over-expression of other *var* genes, which were not represented on the microarray due to sequence diversity. Second, along side with the *var2csa*, a significant number of genes were over-expressed by placental parasites, including genes predicted to encode exported proteins and thus possibly contributing to parasite placental tropism. For this whole-genome analysis of *in vivo* transcription of placental parasites, stringent selection criteria were used i.e. differential expression in at least 2 out of 3 pooled samples after stringent Bonferroni p value adjustment. A common reference containing all parasite erythrocytic stages with an emphasis on schizonts and trophozoites, forms predominantly found in placental isolates, was used. Although there are clearly defined morphologic differences between the schizont, merozoite, and ring stages, biological processes may be shared in contiguous stages. Using a common reference was preferred to three separate controls attempting to match the composition of each pool, as such matching on morphological criteria is very difficult on late stages, if not impossible. This allowed identification of 38 and 46 genes over- or under-expressed in placental parasites respectively.

Of special interest is gene PFI1785w that was highly and specifically over-expressed in placental parasites. The transcription of PFI1785w displayed a clearly defined profile, similar to that of *var2csa*, suggesting a specific association with placental parasites. However, no evidence of its relation to the CSA binding phenotype was seen in parasite lines biologically selected for *in vitro* CSA binding. Several other genes were also over-expressed in placental parasites, but not in *in vitro* selected CSA binding parasites. This suggests either that these genes are selectively over-expressed during pregnancy but are not directly involved in CSA binding, or that conditions required for *in vivo* binding to proteoglycans expressed by the placenta and *in vitro* binding to CSA differ. A recent microarray study on individual parasites by Francis et al. [20] also observed high transcription of PFI1785w by placental parasites, and the expression of this particular PHIST gene at the protein level was described by Fried et al. [21] in membrane extracts from placental parasites. Although CSA binding property represents the major specific phenotype of placental parasites that can be reproduced in laboratory-adapted parasite lines *in vitro*, some characteristics of parasites causing PAM might require *in vivo* conditions for expression. We hypothesise that this could be the case for PFI1785w expression.

The presence of a stop codon (...KKP-stop-KKPHP...) within the 3D7 PFI1785w explains why the gene was miss-annotated as an atypical three–exon PHIST gene and suggests that the sequence downstream the stop codon is not necessary for 3D7 survival *in vitro*. However in other strains such as those causing PAM the entire protein structure may be needed for placental tropism of the parasite.

The recombinant protein (NP561) corresponding to part of the protein encoded by this gene was more highly recognized by serum samples from women living in malaria-endemic areas as compared to serum from men. The relation to gender was less strong than that of VAR2CSA, raising the question of this gene's exact role in the mechanisms governing *P. falciparum* placental tropism. However, the importance of this relation was similar to the one observed with recombinant PFD1140w in the Francis et al. study [20], and the presence of significant levels of antibodies to VAR2CSA has been reported among some men and children [22]. Whether antibodies to PFI1785w express functional activity against PAM parasites will be addressed in future studies. Differential acquisition of these antibodies between men and pregnant women should also be confirmed in a dedicated seroepidemiological survey.

PFI1785w is a single copy gene that encodes a small protein likely exported to the red blood cell as judged by its predicted signal sequence and export motif. The gene is found conserved (>98%) at the nucleotide level in all 4 species of Plasmodium spp infecting humans as well as in the *Plasmodium reichenowi* genome, suggesting that the product of this gene plays an important role for the survival of *Plasmodium*, but argues against a direct role in antigenic variation on the surface of the infected red blood cell. No evidence has been presented that the PHIST proteins in general are surface antigens, and none of them have TM domains. In contrast to PfEMP1 and RIFIN proteins, they exhibit no amino acid diversity between isolates; for example, comparison of PFI1785w alleles between 3D7 and HB3 (Broad Institute browser), the Ghanaian isolate (Sanger browser) or placental isolates from our study shows essentially 100% sequence conservation.

A recent study identified a ∼22 KDa protein as being involved in the binding of iEs to CSA [23]. In the present study, high transcription of a number of genes encoding low molecular weight proteins with predictive characteristics for red blood cell trafficking was specifically observed in placental isolates. Notable examples include, along with PFI1785w (44 KDa corrected here), PFA0700c (12.83 KDa), PF14_0757 (24.95 KDa), and PFB0105c (35.3 KDa). In our study, PF14_0757 with its theoretical molecular weight of 24.95 KDa is the closest in size to this potential protein, but its transcription was not shown to significantly differ between placental and non-placental parasites. Among the 5 genes found by Francis et al. [20] to be specifically co-transcribed with *var2csa* by placental parasites when analyzing individual isolates, four were also identified in our study (PFI1785w, PFL0050c, PFD1140w, PFB0115w). However only PFI1785w met our own criteria of interesting genes (high expression in at least 2 out of the 3 test pools). PFD1140w, the main candidate gene characterized in the Francis et al. study [20] was also detected here, but only in the pool with domination of trophozoites. This apparent discrepancy suggests a stage-dependent expression of the PFD1140w gene. Proteins from 2 (PFI1785w and PFA700c) genes with interesting scores in our study were also detected by Fried et al [21]. For 3 other genes (PFB0115w, PF14_0016, PF14_0260) for which proteins were detected in the same study, differential expression was only recorded in 1 of the 3 test pools. With PF14_0016 and PF14_0260 rather showing PAM under-expression in our study.

Our results suggest that many sub-telomeric genes in *P. falciparum* genome play a role in host-parasite interactions. As sub-telomeric genes, PHIST appears dissimilar to any known structure, making it difficult to assign possible functions to members of this family. A published microarray study of field isolates has shown that most genes from PHISTb and PHISTc families are over-expressed in *P. falciparum* parasites directly collected from infected children compared to 3D7 [24]. As shown in table S2, in the present study, 4 genes belonging to the three PHIST families, i.e. PF14_0757, PFA110w, PFI1785w and PFB105c were found over-expressed in PAM parasites compared to 3D7. Thus, it is all the more interesting that proteomic data support consistent presence of proteins belonging to PHIST family in iE membrane preparations [21], [25], [26], [27] **.**

These findings suggest that other parasite proteins beside VAR2CSA may contribute to the pathogenesis of PAM. Prospective clinical studies are needed to estimate the importance of those new proteins in the mechanism controlling acquisition of distinct binding and serological phenotypes by *P. falciparum* and thus, identification of critical targets for intervention.

## Materials and Methods

### Patients, sample collection and storage

Samples were collected in November 2003 in Guediawaye, a suburb of Dakar, Senegal. The human experimentation guidelines of both French and Senegalese governments were followed. Women were explained the nature of the project, and informed verbal consent was obtained in the presence of an external and independent witness. The study design, the sampling protocol, as well as the way to collect informed consent were approved by the ethical committee of the Ministry of Health, Senegal. Notably verbal consent was considered valid and recommended in this context of poorly literate study population. Pregnant women, admitted for delivery, were enrolled when blood smears and/or immuno-chromatography test were positive for *P. falciparum* infection. Non-pregnant women who presented with fever and whose blood smears were positive for *P. falciparum* infection, were also enrolled as controls. Clinical data were registered and 10 ml of peripheral blood were collected in sodium heparin. Peripheral ring stages iEs were matured for 18–20 h at 37°C in a candle jar [28]. Placental parasites were obtained by perfusing the placenta with 0.1% sodium heparin in PBS [29]. Parasites were conserved in Trizol (Invitrogen) and stored at −80°C, or as spots on Whatman 3MM filter that were dried and stored at room temperature, until subsequent RNA and DNA extractions. Plasma was separated from the peripheral blood and stored at −20°C until use.

During the same period, venous blood samples were collected from asymptomatic infected children in a paralleled on-going cross-sectional study conducted in the neighboring area of Thiès [30]. Samples were collected following the same protocols described above.

### Parasite culture and selection for specific phenotypes

*P. falciparum* strains were cultured as described [31]. The 3D7 strain was sorbitol-synchronized 4 times with an interval of 8 hours to obtain tightly synchronous stages. Strains FCR3 and HB3 were previously panning-selected for adhesion to CD36 or Bewo cells as described [32]. The NF54 strain was selected using antibodies specific to the *var2csa*-encoded PfEMP1 as described [33]. The 3D7 was also panning-selected for adhesion to immobilized CSA. After selection, parasites were cultured 5–6 cycles to generate sufficient parasite density. Mature stages were enriched by Macs purification, as described [34]. *In vitro* panning-selected lines were characterized by reactivity with IgG from multiparous women and lack of reactivity with IgG from *P. falciparum*-exposed men [6], [9], [35].

### DNA and total RNA extractions

Genomic DNA was extracted from filter spots using the chelex procedure [36]. Total RNA from iE containing trophozoite/schizont stage parasites from placental and matured peripheral blood was prepared using organic extraction from Trizol. RNA quality was assessed with Agilent 2100 Bioanalyser.

### Microarrays

The microarrays used here are described elsewhere [12]. Briefly, glass slides were spotted with 8870 70-mer oligonucleotides originating from the Malaria Oligo Set (Qiagen-Operon) and custom design oligonucleotides, covering most of all *P. falciparum* genes. Of the 5542 identified genes, the array covers 5396 genes (97.4%) including protein coding, rRNA, tRNA, snRNA and pseudogenes. Due to the variable distribution of late stages in different isolates after placental perfusion (thin smears reads against 200 parasites), RNA samples from 18 women were pooled into 3 groups. The 3D7 reference was made-up of 10% rings, 45% trophozoites and 45% schizonts. Integrity of the RNA transcripts from each test sample was assessed with an Agilent 2100 Bioanalyser. RNA samples labelling and hybridization were performed as described previously [12].

For each of the three pools of placental parasites, two dye swaps were performed to compensate dye effect and to assess technical reproducibility, leading to four hybridized slides. Data from each of the three pools were analyzed separately using R function (The R project) and Bioconductor package [37] as described previously [12]. After normalization, a paired Student *t* test was used to assess differentially expressed spots and the raw *p* values were then corrected using the Bonferroni method with a type 1 error of 0.05. Log2 ratios of pooled placental parasites versus 3D7 pooled reference was obtained for genes which were found differentially transcribed in at least 2 of 3 test pools and presented in S2. Our data have been submitted to the publicly available ArrayExpress database with the following accession number: E-MEXP-1087.

### Primer design and quantitative real time rt-PCR

Gene-specific real time PCR primers were made based on the 3D7 sequences for 13 selected genes (**supplement Table S1**). Primer amplification efficiency was tested on serial dilutions of gDNA from three laboratory isolates (3D7, FCR3, and HB3) and three different field isolates. Melting curve analysis and agarose gel electrophoresis ensured specificity. The *var2csa* specific primers used are described previously [4].

For real-time PCR, RNA Trizol extraction was followed by 15 min DNase 1 (Sigma) treatment at 37°C. Absence of DNA contamination was assessed by 40 cycles of real time PCR using primers targeting a previously validated endogenous control gene (fructose-biphosphate aldolase) [13]. DNA-free RNA was reverse transcribed using random hexamer primers with Superscript II enzyme (Invitrogen) at 25°C for 10 min, and 42°C for 50 min followed by 70°C for 15 min.

Quantitative real time PCR was performed on cDNA using a Rotorgene thermal cycler system (Corbett Research) [13]. Reactions were done in 20 µl volumes using Quantitect SYBR Green PCR Master Mix (Qiagen) and 0.5 mM primers [13]. Quantitative analysis of the gene-expression level was done using Rotorgene software version 4.6. Transcripts abundances were compared by ΔCt values calculated using fructose-biphosphate aldolase as endogenous control.

Differences between groups of samples studied by real-time rt-PCR were tested by the appropriate non-parametric test (Mann-Whitney two samples rank sum test). Correlations were tested by the Spearman rank sum test. The significant limit was P = 0.05. STATA software (version 7.0, STATA Corporation) was used.

### Sequencing and recombinant proteins generation

The PlasmoDB predicted protein encoded by PFI1785w in the *P. falciparum* clone 3D7 genome, was selected for expression as a soluble protein. The product of this gene is predicted to be exported according to Sargeant et al. [18]. Primers were designed to amplify and clone the full size cDNA and also gDNA, and a fragment of 672 bp from the start codon in order to contain both predicted introns in the 3D7 gene. The forward primer used in the generation of the recombinant protein, NP561, was designed downstream of the apparent stop codon, to avoid a potential un-spliced stop. The sequence from 391 bp to 967 bp was amplified from 3D7 gDNA using the following primers that included EcoR1 and Not1 restriction sites in 5′ and 3′ respectively. Fw: CAATCAAGATATAATAGATCA; Rv: CGGTTTTACCCTTATAATG. All inserts were cloned into a modified pAcGP67A vector containing EcoR1 and Not1 restriction sites, and recombinant proteins produced in *baculovirus*-infected insect cells and purified as described [15]. The purified recombinant protein named NP561, corresponding to about 70% of the full size of the protein was subsequently used as antigen in this study.

### Enzyme-linked Immunosorbent Assay

Plasma levels of *P. falciparum*-specific IgG were measured in ELISA using recombinant VAR2CSA (DBL5ε) or NP561 protein for coating. GLURP recombinant protein was used as control antigen. Two panels of plasma samples from *P. falciparum* thick blood smear negative Ghanaian individuals (30 women of various gravidity and 30 sympatric males) exposed to hyperendemic and seasonal malaria transmission were used [15]. Sera from the two groups were previously characterized for their sex-specific and parity-dependent recognition of VAR2CSA DBL5ε and CSA-adherent parasites [15]. A pool of plasma samples from unexposed Danish women was used as a negative control. Antibody levels were analyzed and expressed as OD values.

## Supporting Information

Table S1 (0.05 MB DOC)

Table S2 (0.35 MB DOC)
