# Supplementary material for: Plasmodium falciparum Transcriptome Analysis Reveals Pregnancy Malaria Associated Gene Expression
Source: PLoS One. 2008 Mar 26;3(3):e1855. doi: 10.1371/journal.pone.0001855 (PMC2267001; doi:10.1371/journal.pone.0001855)
Supplement: Table S1 — (0.05 MB DOC) [file pone.0001855.s001.doc]

Table S1: Supplementary data on the 13 genes quantified by real-time rt-PCR

| **Gene ID** | **Forward primer** | **Reverse primer** | **Array criteria** | **MW** | **Remark** | **description** |
| --- | --- | --- | --- | --- | --- | --- |
| PFA0700c | TTCAGCCTTGAACAAATAACTCA | TTGGTTGTGCCATTGAATTT | 3 out of 3 | 12,8 | export | hypothetical protein conserved in *P. falciparum* |
| PFI1785w | GGTTCTGATGATATGGGTCGT | TCCAAACGTTCCCATGTAGA | 3 out of 3 | 44,0 | export | hypothetical protein |
| PF14_0757 | TGGTCACCTGCTTTGGGTAT | TGCAACACCAATATCATCTTTTG | 3 out of 3 | 25,0 | export | hypothetical protein |
| PFB0105c | TGGTGGAAATGTTGTGGTCA | TGTTGTCCATGAATGCTTTATCA | 2 out of 3 | 35,3 | export | hypothetical protein conserved in *P. falciparum* |
| PF10_0351 | TGCATCTTCCGAGGAAATTA | AATGCATTTCATTTAAATTGTTTGT | 3 out of 3 | 65,1 | tm, ss | hypothetical protein |
| PF14_0010 | AAACTAGCGCAGACCCAGAA | GATCAGCTGCGTAGGCTTTC | 3 out of 3 | 34,6 | export | glycophorin binding protein-related antigen |
| PF10_0344 | AAAATGCCCACGAAACTGTC | CATTTTCAGCTGGTTCATGC | 3 out of 3 | 141,0 | tm, ss | glutamate-rich protein |
| PFC0110w | GCGAAAATAAAAACGAAAATGC | TTCCTTGTTTAAGGAGAGGATATTTT | 3 out of 3 | 167,5 | tm | cytoadherence linked asexual protein CLAG |
| PFD1120c | TACGTTTTTGCCCTCCTCAT | TTTTTCTTTTGCATCTCGTCAA | 3 out of 3 | 14,9 | tm | integral membrane protein conserved in *P. falciparum* |
| PF10_0350 | TGTGTTGAGTGATGCAGAACA | CGGGGTCACTCAAATCATCT | 2 out of 3 | 82,1 | tm | hypothetical protein |
| PFL0260c | CCCTTGGAAGTGCAATAAACA | GCACGATTCGGTATACCTTGA | 2 out of 3 | 39,2 |  | hypothetical protein |
| PFL0030c | AGCCCAATCGGAAGGTAAGT | TTCATAGCTTCTAGCGCCTT | 3 out of 3 | 355,2 | export | var2csa |
