# Supplementary material for: Plasmodium falciparum Transcriptome Analysis Reveals Pregnancy Malaria Associated Gene Expression
Source: PLoS One. 2008 Mar 26;3(3):e1855. doi: 10.1371/journal.pone.0001855 (PMC2267001; doi:10.1371/journal.pone.0001855)
Supplement: Table S2 — (0.35 MB DOC) [file pone.0001855.s002.doc]

**Table S2: Ratio of differentially expressed genes in the 3 pools of placental parasites**

| **gene.ID** | **description** | **pool 1 ratio** | **pool 2 ratio** | **pool 3 ratio** | **tm** | **chr position** | **export** | **family** |
| --- | --- | --- | --- | --- | --- | --- | --- | --- |
| PFC0110w | Cytoadherence linked asexual protein CLAG | 40,97 | 21,55 | 19,88 | 0 | subtel | no | clag |
| FCR3_PFL0030c | var::FCR3 PFL0030c (var2csa) | 10,67 | 39,08 | 12,11 | 1 | subtel | yes | var |
| AS04 | var::ITG2varCS2 5AF134154 | 17,03 | 5,75 | 24,59 | 1 | NA | yes | var |
| PFI1785w | hypothetical protein | 7,08 | 35,45 | 4,63 | 0 | subtel | yes | PHISTb |
| PFC1110w | var::VARC pseudogene | 19,92 | 15,78 | 4,47 | 0 | subtel | NA | var |
| MAL13P1.353 | hypothetical protein | 12,12 | 88,73 | 9,67 | 0 | NA | NA |  |
| PFI0040c | var::VARC-like pseudogene | 5,21 | 19,09 | 4,46 | 0 | subtel | no | var |
| PF07_0131 | var::VAR pseudogene | 6,35 | 13,73 | 4,10 | 0 | subtel | no | var |
| PF10_0351 | hypothetical protein | 5,50 | 12,89 | 3,43 | 0 | cent | no |  |
| PFA0700c | hypothetical protein conserved in P. falciparum | 5,61 | 12,74 | 3,10 | 1 | subtel | yes | hyp10 |
| PF11_0002 | hypothetical protein | 7,92 | 7,12 | 4,18 | 0 | subtel | no |  |
| PF10_0344 | glutamate-rich protein | 8,36 | 3,17 | 2,63 | 0 | cent | no |  |
| MAL6P1.312 | var::erythrocyte membrane protein 1 (PfEMP1) truncated | 3,34 | 6,91 | 3,51 | 0 | subtel | no | var |
| chr14.rRNA-3-5s | 5s rRNA | 5,05 | 3,08 | 5,47 | 0 | cent | NA |  |
| PF14_0010 | glycophorin binding protein-related antigen | 4,93 | 3,96 | 3,89 | 1 | subtel | yes | gbp130 |
| PF13_0353 | NADH-cytochrome b5 reductase putative | 3,83 | 4,12 | 4,35 | 1 | cent | no |  |
| PF14_0757 | hypothetical protein | 3,30 | 2,88 | 4,69 | 1 | subtel | yes | PHISTa |
| PFD0085c | ATP-dept. acyl-coa synthetase putative | 3,86 | 3,15 | 2,93 | 1 | subtel | no |  |
| PF11_0475 | hypothetical protein | 3,21 | 3,04 | 2,67 | 1 | cent | no |  |
| MAL13P1.354 | var::erythrocyte membrane protein 1 (PfEMP1) pseudogene | | 57,35 | 5,25 | 0 | NA | NA | var |
| PFL2585c | RIFIN | 40,34 | 8,99 |  | 2 | subtel | yes | rifin |
| PF13_0008 | erythrocyte membrane protein 1-like | | 17,39 | 3,89 | 1 | subtel | NA |  |
| PFE0015c | degenerate undefined product |  | 9,14 | 3,61 | 0 | subtel | yes | rifin |
| PFI0675w | hypothetical protein | 7,47 | 2,36 |  | 0 | cent | no |  |
| PFB0105c | hypothetical protein | 6,72 | 2,55 |  | 1 | subtel | yes | PHISTc |
| PFA0110w | ring-infected erythrocyte surface antigen precursor RESA | 5,92 | 2,83 |  | 0 | subtel | yes | PHISTb_dnaj |
| PF14_0183 | RNA helicase putative | 3,85 |  | 4,63 | 0 | cent | no |  |
| AS05 | var::ITO R29 |  | 4,56 | 3,60 | 1 | NA | NA | var |
| PFL0315c | hypothetical protein | 4,20 |  | 3,48 | 0 | cent | no |  |
| MAL6P1.211 | hypothetical protein |  | 5,02 | 2,35 | 0 | cent | no |  |
| AS06 | var::ITO4 A4 var | 4,38 |  | 2,75 | 1 | NA | NA | var |
| PF11_0494 | hypothetical protein |  | 3,28 | 3,50 | 0 | subtel | NA |  |
| rRNA_5S | rRNA_5S ribosomal RNA gene | 3,14 |  | 2,71 | 0 | cent | NA |  |
| PFA0760w | RIFIN |  | 3,03 | 2,58 | 1 | subtel | yes | rifin |
| PFB0995w | hypothetical protein | 3,15 | 2,41 |  | 2 | subtel | yes | hyp5 |
| PF14_0153 | hypothetical protein | 0,29 | 0,34 | 0,38 | 4 | cent | no |  |
| PFC0885c | hypothetical protein | 0,31 | 0,39 | 0,31 | 0 | cent | no |  |
| MAL13P1.257 | hypothetical protein conserved | 0,32 | 0,39 | 0,25 | 0 | cent | no |  |
| PFE0685w | hypothetical protein | 0,30 | 0,38 | 0,26 | 7 | cent | no |  |
| PF11_0414 | hypothetical protein | 0,31 | 0,37 | 0,21 | 0 | cent | no |  |
| PF14_0151 | hypothetical protein | 0,32 | 0,36 | 0,18 | 0 | cent | no |  |
| PFD0595w | hypothetical protein | 0,27 | 0,39 | 0,18 | 2 | cent | no |  |
| PF14_0697 | dihydroorotase putative | 0,32 | 0,30 | 0,20 | 0 | cent | no |  |
| chr8.rRNA-2-28s-pseudo | 28s pseudo | 0,22 | 0,34 | 0,17 | 0 | NA | NA |  |
| chr5.rRNA-1-ITS1-A | ITS1 A-type | 0,24 | 0,24 | 0,18 | 0 | subtel | NA |  |
| PF14_0744 | hypothetical protein | 0,25 | 0,26 | 0,12 | 0 | subtel | yes | unique |
| PFD1120c | integral membrane protein conserved in P. falciparum | 0,26 | 0,25 | 0,12 | 2 | subtel | no |  |
| chr7.rRNA-1-ITS1 | ITS1 putative | 0,19 | 0,24 | 0,18 | 0 | NA | NA |  |
| PFL1155w | GTP cyclohydrolase I | 0,19 | 0,16 | 0,22 | 0 | cent | no |  |
| PFD0310w | stage-specific protein precursor sexual stage-specific protein precursor | 0,17 | 0,20 | 0,13 | 2 | cent | no |  |
| chr8.rRNA-1-5.8s-pseudo | 5.8s pseudo | 0,06 | 0,17 | 0,02 | 0 | NA | NA |  |
| PF08_0085 | ubiquitin-conjugating enzyme putative | | 0,42 | 0,37 | 0 | cent | no |  |
| PF14_0020 | choline kinase putative |  | 0,42 | 0,36 | 0 | subtel | no |  |
| PF07_0049 | var::erythrocyte membrane protein 1 (PfEMP1) | | 0,41 | 0,36 | 0 | cent | yes | var |
| PFL2460w | coronin |  | 0,33 | 0,44 | 0 | cent | no |  |
| MAL6P1.302 | hypothetical protein |  | 0,38 | 0,37 | 3 | cent | no |  |
| PFL0180w | cytochrome c1 heme lyase putative | | 0,36 | 0,38 | 0 | cent | no |  |
| PFI0405w | hypothetical protein |  | 0,38 | 0,36 | 2 | cent | no |  |
| MAL6P1.202 | hypothetical protein |  | 0,42 | 0,30 | 5 | cent | no |  |
| PF10_0323 | hypothetical protein | 0,28 |  | 0,44 | 2 | cent | no |  |
| PFD1020c | RIFIN |  | 0,30 | 0,41 | 1 | cent | yes | rifin |
| PF11_0309 | hypothetical protein | 0,27 |  | 0,43 | 2 | cent | no |  |
| PFE1410c | hypothetical protein |  | 0,39 | 0,28 | 0 | cent | no |  |
| PFL1870c | hypothetical protein |  | 0,40 | 0,27 | 0 | cent | no |  |
| PF10_0141 | cdk7 putative | 0,33 |  | 0,33 | 0 | cent | no |  |
| PFL0795c | hypothetical protein |  | 0,27 | 0,38 | 0 | cent | no |  |
| PF11_0172 | hypothetical protein conserved | | 0,37 | 0,27 | 11 | cent | no |  |
| PFA0565c | hypothetical protein | 0,33 |  | 0,25 | 0 | cent | no |  |
| PFL0260c | hypothetical protein |  | 0,34 | 0,23 | 0 | cent | no |  |
| PFL0750w | hypothetical protein |  | 0,25 | 0,29 | 0 | cent | no |  |
| PF14_0187 | glutathione s-transferase putative | 0,31 |  | 0,21 | 0 | cent | no |  |
| PF10_0350 | hypothetical protein |  | 0,32 | 0,17 | 0 | cent | no |  |
| chr13_2.rRNA-28s-S | 28s rRNA S-type |  | 0,32 | 0,14 | 0 | NA | NA |  |
| chr11.rRNA-1-18s-S | 18s rRNA S-type |  | 0,27 | 0,14 | 0 | NA | NA |  |
| MAL13P1.245 | hypothetical protein |  | 0,19 | 0,22 | 0 | cent | no |  |
| PF13_0347 | hypothetical protein | 0,23 |  | 0,15 | 0 | cent | no |  |
| PF14_0745 | hypothetical protein |  | 0,19 | 0,14 | 0 | subtel | no |  |
| PF14_0433 | hypothetical protein | 0,22 |  | 0,10 | 0 | cent | no |  |
| PF11_0490 | hypothetical protein | 0,25 |  | 0,05 | 1 | NA | NA |  |

Pool 1 included 3 isolates (< 5% rings, 70% trophozoites, >25% schizonts); Pool 2 with 7 isolates (< 5% rings, >25% trophozoites, 70% schizonts); Pool 3 with 8 isolates (< 5% rings, 0% trophozoites, >95% schizonts) versus a common 3D7 reference pool (10% rings, 45% trophozoites, 45% schizonts). Only genes with statistically significant ratios are shown (p-value <0.05 after adjustment by the Bonferroni procedure). In case of two or more probes per gene, mean ratio is represented. Sub-telomeric genes are defined as genes within the first 150kb from the telomers. Are also shown TM prediction (PlasmoDB) and export prediction (Sargeant et al., 2006). Genes families are defined as in Sargeant et al. (2006), in addition to *var* and *clag* families.
